# Day-to-day variation of the kidney proximal tubular injury markers urinary cystatin C, KIM1, and NGAL in patients with chronic kidney disease

**DOI:** 10.1080/0886022X.2020.1757463

**Published:** 2020-04-29

**Authors:** Inga Soveri, Johanna Helmersson-Karlqvist, Bengt Fellström, Anders Larsson

**Affiliations:** Department of Medical Sciences, Uppsala University, Uppsala University Hospital, Uppsala, Sweden

**Keywords:** U-KIM1, U-NGAL, U-cystatin C, Day-to-day variation, proximal tubular injury markers

## Abstract

**Background:**

It is important to know the intraindividual variation of biomarkers to be able to distinguish a change of a biomarker due to the course of the disease from the normal biological variation of the marker. The purpose of this study was to investigate the day-to-day variability of urine markers in nephrology patients.

**Materials:**

23 nephrology patients were included in the study. First morning urine samples were collected daily for ten consecutive days and analyzed for U-cystatin C, U-KIM1, U-NGAL and U-creatinine. The day-to-day variation was calculated as concentrations of the markers and as creatinine ratios. Values deviating more than the 90^th^ percentile of the normal intraindividual variation was used to define a disease/treatment specific change.

**Results:**

The day-to-day coefficient of variation (CV) for individual patients varied between 9.6 and 100.3% for NGAL (mean 45.6%) and between 8.8 and 107.3% for the NGAL/creatinine ratio (mean 43.8%). The corresponding values for KIM1 were between 10.9 and 60.2% (mean 30.1%) and for the ratio between 8.7 and 59.8% (mean 23.4%) and for cystatin C 3.8–67.4% (mean 25.0%) and for the cystatin C/creatinine ratio 5.9–78.4% (mean 24.8%).

**Conclusions:**

The similar intraindividual CV values between the renal tubules damage markers and their corresponding creatinine ratios speaks against using creatinine ratio. Using the 90^th^ percentiles of the CV values as a limit for clinical change means that NGAL has to change by 83.3%, KIM1 by 45.5% and Cystatin C by 46.3% before the change can be considered clinically significant in patients with chronic kidney disease.

## Introduction

It is essential to distinguish between the change of a biomarker due to the course of the disease and/or due to the treatment as opposed to change because of nonspecific variation. It is thus important to be aware of the normal variation of biomarkers in the patient population. The variation not related to the specific disease is due to preanalytical, analytical and postanalytical variation [1–3]. Analytical variation is a combination of analytical imprecision and bias [4]. The analytical imprecision is routinely monitored by the hospital laboratories using internal control materials. Most laboratories also participate in external quality assurance programs and the results from these programs can be used to monitor bias in relation to the other laboratories participating in the same program. The preanalytical variation is much more complex and is dependent on a number of factors related to the patient and to the collection of the sample. Patient factors include for instance the time of sampling, food intake, exercise, medication, urine volume, muscle mass and level of the analyte [5]. Several of these factors differ between patient groups and healthy controls. Most studies on intraindividual variations are performed in healthy individuals, most likely due to the fact that there are a great number of diseases and it is difficult to cover all diseases. It is easier to study the intraindividual variation if the biomarker is mainly used by a specific patient group because the study can include only this patient group. The use of kidney tubular injury markers are typical examples of markers that are almost exclusively used in patients with known or suspected kidney diseases.

This study focused on the day to day variation of three markers for proximal tubuli damage [6–9]. Many conditions may cause tubular injury including diabetes, infections, surgery, heart failure, burns, hypercalcemia and administration of drugs such as contrast media, antimicrobials, chemotherapeutics, analgesics and immunosuppressives [10–12]. Cystatin C is a small (13.3 kDa) non-glycosylated protein that is freely filtered in the kidney glomerulus and then efficiently reabsorbed and catabolized in the proximal tubular cells. In healthy individuals the urinary concentration of cystatin C is low while increased levels indicate a functional defect in the proximal tubular cells [13]. NGAL and KIM1 are also markers of damage of the proximal tubular cells. In contrast to cystatin C, NGAL and KIM1 are expressed in tubular epithelial cells after injury. This means that NGAL and KIM1 are markers for acute/ongoing injury leading to increased expression of the markers in the tubular cells while cystatin C is also reflecting an older injury leading to a decreased function of the cells.

Today, the majority of urine proteins are measured in spot urine or first morning urine samples rather than 24 h collections. Many laboratories report the results of the urine markers as creatinine ratios [14]. This is mainly based on a tradition developed for urine albumin. The use of creatinine ratios for tubular damage markers are less well documented [14–17].

The aim of the present study was to study the day-to-day variation of U-cystatin C, U-KIM1 and U-NGAL in first morning spot urine samples as concentrations and as creatinine ratios. We included only patients with stable chronic kidney disease in order to minimize the effects of improvements/deterioration of the underlying disease. The study period was limited to ten consecutive days to reduce the effects of disease changes over time.

## Methods

### Patients

Clinically stable adult outpatients followed at the department of nephrology, Uppsala University Hospital, Uppsala, Sweden, were asked to participate in the study. The study was approved by the regional ethics review board at Uppsala University (2016/40). Treatment with immunosuppressive medicines was the exclusion criterion. Written informed consent was obtained from all patients prior to enrollment. Twenty-three patients were included in the study. Urine samples were collected daily as first morning urine samples for a total of ten samples per patient. The samples were initially frozen at −20 by the patient and then transferred to a −70 freezer for long time storage. The samples were analyzed within 6 months from collection. Prior to analysis the urine samples were thawed and centrifuged at 1500 g for 10 min at ambient temperature.

### Assays

U-Cystatin C and U-Creatinine were analyzed on a BS380 instrument (Mindray, Shenzhen, China). The creatinine reagents were from Abbott Laboratories (Abbott Park, IL, USA) and the cystatin C reagent was from Gentian (Moss, Norway). The total coefficient of variation (CV) were 2.5% at 0.48 mg/L and 1.5% at 0.8 mg/L for U-Cystatin C and 1.4% at 8.5 mmol/L and 1.5% at 4.2 mmol/L for U-Creatinine. eGFR_Cr_, in mL/min/1.73m^2^, was estimated using the revised Lund-Malmö equation [18].

U-NGAL and U-KIM1 were analyzed were analyzed by commercial sandwich ELISA kits (DY1757 and DY1750B, R&D Systems, Minneapolis, MN, USA). The assays were calibrated against highly purified recombinant human peptides and the total CV of the assays were approximately 6%. All assays were performed blinded without knowledge of the clinical diagnosis.

### Statistical analysis

Calculation of coefficients of variations were performed with Statistica (StatSoft, Tulsa, OK, USA). U-NGAL values below the lowest standard point in the ELISA were excluded in the analysis of intraindividual variation. This meant that two individuals were excluded when calculating variation for NGAL and the NGAL/creatinine ratio. Pearson linear correlations were used for studying log-log associations between the concentrations of the studied markers and creatinine values in individual patients. To address the effect of 23 measurements for each marker the limit for significance was adjusted to a *p*-value of 0.002. Performing a large number of tests increases the risk of false discoveries. Therefore, we adjusted the P values for multiplicity testing by dividing 0.05/23 = 0.002.

## Results

### Patient characteristics

The study population consisted of 18 males and 5 females. The mean age was 68 years (range 49–84 years) and the mean creatinine estimated GFR was 38.7 mL/min/1.73m^2^ (range 9–90 mL/min/1.73m^2^). Patient characteristics are presented in Table 1.

**Table 1.** Basic data for the patients participating in the study.

Patient	No of samples	Gender	Age (years)	eGFR	Diagnose
1	10	Male	72	53	Polycystic kidney disease
2	10	Male	49	16	Type 1 diabetes mellitus
3	10	Female	55	90	Alport syndrome
4	10	Female	54	79	Polycystic kidney disease
5	10	Male	73	13	Hypertensive kidney disease
6	10	Male	61	26	IgA nephropathy
7	10	Male	77	57	IgA nephropathy
8	10	Male	73	50	Post-Streptococcal glomerulonephritis
9	10	Female	76	53	Leucocytoclastic vasculitis
10	10	Male	76	9	Hypertensive kidney disease
11	10	Male	84	36	Hypertensive kidney disease
12	10	Male	82	37	Hypertensive kidney disease
13	10	Male	64	28	Chronic tubular damage, hypertension
14	10	Female	66	36	IgA nephropathy
15	9	Male	69	19	Post-interstitial nephritis
16	10	Male	77	28	Type 2 diabetes mellitus
17	9	Male	72	49	Hypertensive kidney disease
18	10	Female	54	54	SLE nephritis
19	10	Male	76	43	Hypertensive kidney disease
20	10	Male	75	24	Hypertensive kidney disease
21	10	Male	53	68	IgA nephropathy
22	10	Male	70	10	Hereditary spherocytosis
23	7	Male	54	11	Polycystic kidney disease

eGFR was calculated from plasma creatinine values.

### Dynamic range of the studied kidney tubular markers

The dynamic range, defined as the ratio between the lowest and highest observed mean value for individual patients, varied considerably. The highest ratio was observed for Cystatin C (194.0) while the corresponding ratios were for NGAL 54.8, for KIM1 6.02 and for creatinine 3.65 (Table 2).

**Table 2.** Mean values for individual patients and ratio between lowest and highest value observed for urinary NGAL, KIM1, Cystatin C and creatinine.

	NGAL	KIM1	Cystatin C	Creatinine
	ng/L	ng/L	mg/L	mmol/L
1	14184	1554	0.06	12.36
2	111402	1091	1.69	4.15
3	7117	817	0.08	8.81
4	7809	1704	0.04	9.68
5	205018	604	2.15	6.45
6	6139	1051	0.11	6.64
7	10306	726	0.13	8.02
8	125996	1697	0.09	12.18
9	25007	610	0.06	8.05
10	336889	1217	7.76	7.19
11	30161	440	0.08	5.83
12	8019	788	0.08	5.88
13	7360	1280	0.08	9.08
14	20065	604	0.09	6.41
15	7052	513	0.21	8.34
16	17037	368	0.33	6.72
17	15067	283	0.07	8.91
18	18125	314	0.06	14.22
19	13802	1216	0.08	14.92
20	47993	298	0.31	7.38
21	71028	500	0.09	14.68
22	178499	304	2.14	4.09
23	269160	375	2.33	5.95
Ratio	54.8	6.02	194.0	3.65

### Variation in U-cystatin C, U-KIM1, U-NGAL and the corresponding creatinine ratios

The interday CV for individual patients varied between 9.6 and 100.3% for NGAL and between 8.8 and 107.3% for the NGAL/creatinine ratio. The corresponding values for KIM1 were between 10.9 and 60.2% and for the ratio between 8.7 and 59.8% and for cystatin C 3.8–67.4% and for the cystatin C/creatinine ratio 5.9–78.4% (Table 3).

**Table 3.** Coefficient of variation (CV) for urinary NGAL, KIM1, cystatin C (Cyst C) and creatinine (crea) and the corresponding creatinine ratios in individual patients.

		NGAL	KIM1	Cyst C	Crea	NGAL/ crea	KIM1/ crea	Cyst C/ crea
*N*	Patient	CV	CV	CV	CV	CV	CV	CV
10	1	42.4	42.0	32.6	30.7	41.1	16.0	33.5
10	2	13.9	12.4	14.4	12.0	8.8	8.7	10.7
10	3	65.3	40.3	23.6	33.7	59.6	24.1	20.6
10	4	48.7	44.8	21.8	23.3	44.1	35.3	34.2
10	5	13.5	16.5	14.7	6.0	10.1	12.0	13.4
10	6	17.2	10.9	16.2	14.3	18.9	12.8	7.8
10	7		19.0	67.4	22.7		24.5	56.0
10	8	92.6	28.4	15.8	20.4	82.7	17.2	7.2
10	9	61.2	26.8	38.9	33.7	43.7	36.4	30.9
10	10	27.0	25.4	12.8	18.1	24.0	9.8	9.5
10	11	100.3	39.4	41.0	29.0	107.3	17.1	56.7
10	12		27.5	28.3	27.5		28.0	11.7
10	13	71.5	29.1	46.3	22.9	46.9	10.9	32.9
10	14	44.7	49.3	12.3	28.4	24.6	20.5	15.4
9	15	27.1	15.6	16.0	14.5	41.5	12.2	19.9
10	16	35.1	25.0	44.2	21.6	47.2	31.2	78.4
9	17	55.7	22.0	14.2	18.0	65.4	18.2	7.6
10	18	43.9	26.0	21.5	13.6	37.5	23.9	18.9
10	19	46.5	45.5	49.8	9.5	52.2	43.1	56.2
10	20	83.3	21.8	14.3	10.3	89.9	24.5	16.4
10	21	29.4	39.9	21.2	13.8	24.5	35.5	18.3
10	22	9.6	60.2	4.7	5.3	9.9	59.8	5.9
7	23	29.6	25.0	3.8	11.7	38.8	16.8	8.9
	mean	45.6	30.1	25.0	19.2	43.8	23.4	24.8

*N*: samples from each patient. Patient 7 and 12 had urinary NGAL values below the lowest standard point in the ELISA and were therefore excluded in the analysis of intraindividual variation.

The 90^th^ percentiles for the CV values were similar for NGAL and NGAL/creatinine ratio (83.3 vs 82.7%), for KIM1 and KIM1/creatinine ratio (45.5 vs 36.4%) and for cystatin C and cystatin C/creatinine ratio (46.3 vs 56.2%).

### Pearson linear correlations between the studied kidney tubular markers and creatinine

A positive linear correlation between the tubular biomarker and U-Creatinine would strengthen the theoretical rationale for using the creatinine ratio. U-Creatinine showed a significant positive correlation with U-KIM1 in five patients. Five patients had significant positive correlations between U-cystatin C and U-creatinine while none of the patients showed significant associations between U-NGAL and U-creatinine.

## Discussion

It is important to be aware of the normal variation between two sampling times to be able to evaluate when a true change has occurred. Both false positive and false negative interpretations may lead to incorrect treatment. The aim of the present study was to investigate the natural variation for these markers in CKD patients. Before we can introduce new tubular damage markers in routine we need to know how to evaluate the results and the day-to-day variation is an important part of the evaluation.

Increased urine volumes reduce the urine protein and creatinine values by diluting the analytes. The creatinine ratio is used to reduce the variation in urine volume as it is believed that the patients creatinine production from the muscles should be more stable over time than the intake of liquids and urine volume.

This study showed that only a minority of patients had positive correlations between the tubular markers U-NGAL and U-cystatin C and U-creatinine which argue against creatinine adjustment. A possible explanation could be that the increase in urine of the tubular markers is associated with reduced tubular function. A reduced tubular function could potentially reduce the tubular secretion of creatinine. Urinary creatinine is mainly derived from glomerular filtration, but there is also a tubular secretion of creatinine that contributes to part of the urine creatinine. In patients with normal renal function tubular secretion accounts for 10-40% of the GFR derived urinary creatinine but secreted creatinine is increased in patients with reduced GFR and may increase to more than 100% of the creatinine produced by glomerular filtration in patients with GFR about 40 mL/min/1.73 m^2^ [19]. None of the three studied tubular markers had a significant positive correlation to the creatinine value indicating that creatinine should not be used to adjust the concentration of these markers in patients with chronic kidney disease. The intraindividual CVs of the different markers and their corresponding creatinine ratios were also fairly similar. The use of creatinine ratios will thus not reduce the day to day variation of these tubular markers. Even if the cost for the U-creatinine assay usually is low, it will still increase the assay cost.

NGAL had the highest intraindividual variation of the three studied tubular damage markers.

The higher dynamic range for cystatin C will partly offset the high CV when trying to decide when there is a significant change. The CVs for U-cystatin C and U-creatinine assays were in the 1–2% range and the CVs for the ELISA methods were approximately 6%. Thus the assay variation only contributed to a very small part of the total CV of the studied biomarkers.

A strength of the present study is that it only included CKD patients rather than healthy controls and that the patients had very well characterized clinical diagnoses. The weakness is that it was a limited number of patients with varying diagnosis. It is thus not possible to analyze the variation in relation to underlying disease or GFR values. We chose to study morning urine samples rather than 24 h urine collections. The longer collection periods for 24 h collections should in theory reduce the day to day variation if the collection is performed correctly. A major problem with 24 h collections is that there are often errors in the sampling period. It is also inconvenient for the patient as the containers are rather bulky, there may be problems with leakage and preferably the samples should be kept refrigerated during the sample period to avoid protein degradation. It can not be expected that the patient will store the urine in their home refrigerator. Therefore, morning urine samples have whenever possible replaced 24 h urine collections.

We used the 90^th^ percentile of the intraindividual variations as a decision point for a clinically important change. The 90^th^ percentile means that one in ten results is outside the expected value due to normal variation. We consider this as an acceptable decision limit to distinguish between normal variation and disease related changes. Using the 90^th^ percentiles of the CV values as a limit for clinical change means that the concentration of NGAL has to change by 83.3%, KIM1 by 45.5% and Cystatin C by 46.3% before the change can be considered clinically significant in patients with chronic kidney disease.
